# Management of Tourniquet-Related Nerve Injury (TRNI): A Systematic Review

**DOI:** 10.7759/cureus.27685

**Published:** 2022-08-04

**Authors:** Jeremy Chang, Laxminarayan Bhandari, Joseph Messana, Saud Alkabbaa, Alireza Hamidian Jahromi, Petros Konofaos

**Affiliations:** 1 Internal Medicine, University of Tennessee Health Science Center, Memphis, USA; 2 Hand Surgery, Kleinert Kutz and Associates, University of Louisville, Louisville, USA; 3 Hand Surgery, Christine M Kleinert Institute of Hand and Microsurgery, Louisville, USA; 4 Plastic Surgery, King Fahad Armed Forces Hospital, Jeddah, SAU; 5 Plastic Surgery, Temple University Hospital, Philadelphia, USA; 6 Plastic Surgery, University of Tennessee Health Science Center, Memphis, USA

**Keywords:** peripheral nerve disorders, nerve palsy, neuropraxia, nerve trauma, tourniquet use

## Abstract

Tourniquet-related nerve injuries (TRNIs) are a rare but feared complication of operative tourniquet use. While the literature contains multiple discussions regarding tourniquet use as well as reported cases of its complications, there does not exist a consensus guideline for a safe tourniquet pressure, application time, or management of TRNI. This paper conducts a comprehensive review of the available literature for cases of TRNI with a specific focus on analyzing the management of cases of TRNI and their functional recovery. One hundred nine articles were retrieved in a search of medical literature (PubMed) using the keywords: tourniquet, nerve injury, paralysis, and palsy. The initial search was further narrowed down to seven case series and 10 case reports totaling 203 reported cases of TRNI. Of the 203 cases, 64 cases involved upper extremity tourniquet use, and 139 cases involved lower extremity tourniquet use. Most patients (89.75%) experienced a complete recovery. TRNI may occur over a wide range of tourniquet application times and tourniquet pressures; hence, it is a necessity for surgeons to consider it as a potential complication and understand the methodology for diagnosis and long-term management.

## Introduction and background

Tourniquets are commonly used in surgeries to minimize blood loss and improve visualization. The term “Tourniquet” is derived from the French word “Tourner” which means “to turn.” Historically, the use of tourniquets dates back to battlegrounds where tourniquets were applied with a piece of clothing or filet to prevent exsanguination following sharp injuries and amputations [1]. Later, other devices were designed for a bloodless surgical field, sharing the same principle: localized pressure over vascular structures supplying the limb to prevent unwarranted blood loss. Louis Petit (1674-1750) described the screw tourniquet device, Johann Friedrich August von Esmarch (1828-1908) reported the use of flat rubber bandages and Harvey Cushing (1869-1939) introduced the pneumatic tourniquet for limb surgeries [1-3]. A modern pneumatic tourniquet consists of three basic parts: 1) A cuff that is wrapped around the patient’s limb, 2) a source of compressed air which helps in creating the compression and 3) a pressure maintenance mechanism including a pressure gauge and controllers to compensate for minor air leaks.

The use of tourniquets is not without complications. These are the inability to adequately control hemorrhage, and injury to the skin, blood vessels, nerves, and muscles including reperfusion injury and rhabdomyolysis, nerve injury, and systemic effects [4]. The most dreaded complication is nerve injury following tourniquet application. Although rare, the implications and long-term sequelae of tourniquet-related nerve injuries (TRNIs) are devastating [4].

Many authors have discussed the pathophysiology of TRNI. Although there are recommendations in the medical literature on how to “prevent’ TRNI, these are inconclusive and conflicting [4,5]. The guidelines should ideally be detailed, and evidence-based and must include information on set pressure, duration of application, interval times, padding, equipment (tourniquet machine) calibration, and controlling patient’s intraoperative blood pressure, etc. There is little consensus regarding tourniquet use and, once TRNI occurs, there is limited data on its management. The aim of this study is to review the literature for existing recommendations on the management of TRNI, to review how TRNI was managed in reported cases, and to analyze the outcome.

## Review

Study design

The study was conducted based on PRISMA 2020 guidelines. Articles were obtained through the US National Library of Medicine (NLM) database, and PubMed. The search was narrowed down by using keywords like “tourniquet,” “nerve injury,” “palsy,” and “paralysis.” We searched for the articles that included the above-mentioned keywords within their titles or abstract fields. A date range was not set. Following an initial search, article abstracts were utilized to categorize the articles into the following categories: “Basic Science,” “Case Reports,” “Case Series,” “Review,” and “Commentary or Letter to Editor.” At this stage, any articles that were unrelated to TRNI were excluded. Articles categorized as “Case Reports” and “Case Series” were further evaluated. 

Inclusion criteria for selected articles were: 1) articles that document the exact number of cases of TRNI, 2) articles that include demographics and documents supporting the diagnosis of nerve injury due to tourniquet use, 3) Articles that report patient management and outcome of TRNI (i.e., complete recovery vs partial recovery vs no recovery), and 4) cases in which a tourniquet was used in an operative setting. 

Articles were excluded from the study if 1) nerve injury did not occur, 2) tourniquet use and associated complications were not documented, and 3) lack of sustained or adequate follow-up (greater than three months or until nerve injury was resolved or stabilized) to documenting the outcome.

**Figure 1:**
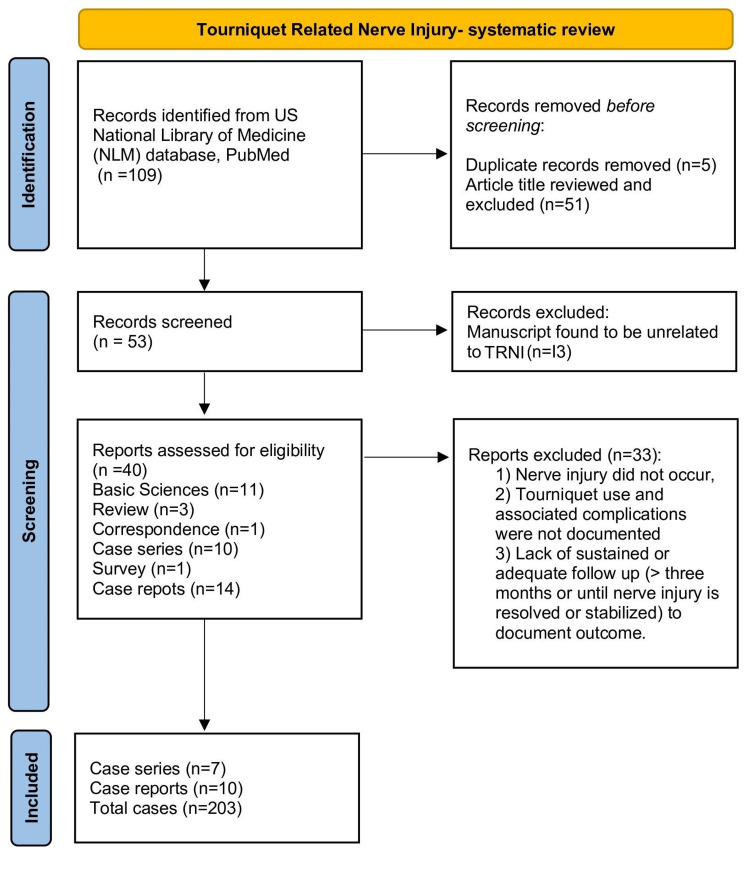
Current study design and algorithm of approach for finding the reported cases with tourniquet-related nerve injury (TRNI) from medical literature as per PRISMA guidelines

Articles were also excluded if they were not available in the English language (utilized ILLiad interlibrary loan system [OCLC, Dublin, OH]). “Basic science articles” and “review articles” were also reviewed for additional input on the background and preparation of detailed discussions (Figure 1).

Additional demographic information was obtained from the data and results of the “case reports” and “case series” in review. These included age, ethnicity, gender, and body mass index where applicable. Furthermore, the duration of tourniquet use, tourniquet pressure, complications documented to tourniquet use, their management, and long-term outcomes were identified. Shared characteristics between cases of TRNI among different study groups were also identified.

Basic summary statistics (i.e., mean, median, mode, range) were used to describe the data. Additionally, Student’s t-test and the chi-square test (χ² test) were used where appropriate. Data, figures, and tables were organized, stored, and analyzed using Microsoft Excel (Microsoft, Redmond, WA).

Results

An initial database search on PubMed revealed 109 articles. After vetting the titles, 53 articles and their abstracts were thoroughly studied. Forty out of 53 articles were found related to TRNI. These articles were further categorized into “Case Reports and Case Series” (N=25), “Basic Science” (n=11), “Review” (n=3), and “Correspondence” (n=1). Twenty-five "case reports and case series” were further categorized into ten case series, one survey, and 14 single case reports. Based on the inclusion and exclusion criteria, seven case series and ten single case reports were reviewed and their analysis yielded 203 cases of TRNI.

Upon further analysis, it was found that a pneumatic tourniquet was used in all except for two - (i) a case series by Akinyoola et al. in which an Esmarch tourniquet was used, and (ii) a case series by Landi et al. in which both the Esmarch and pneumatic tourniquet were used [6,7]. There was no mention of the use of elastic exsanguination in any of the studies. Among the 28 cases where gender was reported, there were 21 male and seven female patients with TRNI. Only 28 cases were found to have reported the exact age of the patients, with five patients ranging between 0 and 18 years (pediatric), 23 patients between 19 and 64 years (adult), and none of the patients above 65 years (senior) (Table 1). Among the 203 cases with TRNI, 64 were reported to have nerve injuries in the upper extremity (UE) while the rest were in the lower extremity (LE) (Table 1).

**Table 1:** Demographic characteristics of the cases with Tourniquet Related Nerve Injury (TRNI) in the medical literature (N=203)

Gender	Male	21
	Female	7
	unrecorded	175
Age	0-18	5
	19-64	23
	>65	0
	unrecorded	175

In the majority of cases with TRNI in the UE, the tourniquet pressure ranged between 251 and 300 mmHg. Many authors did not report the tourniquet pressures in LE TRNI, since most cases originated from a large case series by Horlocker et al. (129 cases) [8]. Horlocker et al. only note that 92% of the LE tourniquet applications in their retrospective case series were recorded at 300 mmHg; however, the pressure of the remaining 8% was not reported. [8]. In addition, TRNIs appear to occur over a wide range of tourniquet application durations (interrupted or uninterrupted tourniquet time). All cases with tourniquet application durations of more than 60 minutes note the use of at least one tourniquet deflation for 4-10 minutes. Tourniquet application durations for UE TRNI cases (n=64) ranged from 28 to 720 minutes, whereas, in LE TRNIs, the duration ranged from 40 to 308 minutes (n=139) (Table 2).

**Table 2:** Characteristics of tourniquet application in the reported cases with tourniquet-related nerve injury (TRNI) reported in the medical literature (n=203)

Factors		Upper Extremity (n=64)	Lower Extremity (n=139)
	201-250	7 (10.9%)	1 (0.7%)
	251-300	35 (54.7%)	1 (0.7%)
Pressure (mmHg)	301-350	0	1 (0.7%)
	>350	0	5 (3.6%)
	unrecorded	21 (32.8%)	131 (94.2%)
	<60	8 (12.5%)	1 (0.7%)
	61-90	9 (14.1%)	0
Duration (min)	91-120	3 (4.7%)	1 (0.7%)
	>120	6 (9.4%)	2 (1.4%)
	unrecorded	38 (59.4%)	135 (97.1%)

Among the 203 reported cases of TRNI, 89.7% (182/203) had a complete recovery, 8.3% (17/203) had a partial recovery and 2% (4/203) had no recovery (Table 3). The four cases of TRNI that resulted in no recovery and had permanent sensory and/or motor deficit resulted from tourniquet application to the LE. Time to complete recovery was described to vary widely, ranging between six days to 18 months post-operatively.

**Table 3:** Distribution of the cases with TRNI (N=203) and their recovery characteristics.

Recovery	Upper Extremity (n=64)	Lower Extremity (n=139)
Complete	56 (87.5%)	126 (90.6%)
Partial	8 (12.5%)	9 (6.5%)
None	0	4 (2.9%)

Management of TRNIs was found to be sporadically discussed in the reported cases. All case reports described the specific use of both electromyography (EMG) and nerve conduction studies (NCSs) to diagnose a sensory deficit. The timing of these diagnostic tests as well as the frequency of repeated testing was found to vary. The first postoperative test was reported most commonly around 8-12 weeks postoperatively. Earlier case reports also described the necessity of ensuring accurate gauge measurements of tourniquet pressure. None of the patients underwent surgical treatment for TRNI.

Discussion

Paralysis of an upper limb after tourniquet use was first described more than 130 years ago by Montes (in a Mexican Journal) and Putnam (in Boston Medical Surgical Journal) [9]. However, the first operative pneumatic TRNI was reported in the literature by Moldaver in 1954 [10].

Since then, there have been few studies investigating the rate of occurrence of TRNIs. In 1974, a survey of 151 orthopedic surgeons by Middleton et al., including an estimated 630,000 applications of tourniquets estimated the overall rate of TRNI to be at one in 8,000, which could be further divided into one in 5,000 in the UE, and one in 13,000 in the LE [11]. A more contemporary analysis of tourniquet use in Norway reported a TRNI rate of one in 6,155 [4].

This wide variation is thought to be due to under-diagnosis of nerve injury, the complexity of the clinical setting, presence of pain at the site of surgery, concurrent limb weakness postoperatively from muscle injury as well as the often-rapid recovery of the affected nerve(s) [12]. Studies have shown a higher frequency of nerve injuries in the upper extremities versus lower extremities, with the radial nerve being more prone to tourniquet-associated injury than the ulnar nerve while the median nerve is the least susceptible [4,7,8,13]. The most commonly injured nerve of the LE is the sciatic nerve [14]. Mechanical pressure seems to be more important in the mechanism of nerve injury than distal ischemia [15].

Analysis of 203 reports of TRNI spanning over 50 years of operative tourniquet use shows it is difficult to establish major shared characteristics between cases. The findings support the notion that TRNI may occur over a wide range of tourniquet application pressures as well as a wide range of application times. Furthermore, although a majority of patients (89.7%) experienced complete functional recovery, the time frame of recovery is highly variable. In this data set, there were more cases of LE TRNI compared with UE TRNI; however, this is primarily due to the larger case series in LE included in the data analysis. 

Although some of the authors have discussed TRNI, there is little high-level evidence to support consensus guidelines regarding tourniquet use and the management of TRNI. Various suggestions for safe tourniquet inflation pressures and management of inflation times exist in the literature. Jullian Bruner originally proposed in 1951, that pressures of 270 to 300 mmHg for adults and 250 mmHg or less for children were appropriate [16]. In 1972, Adrian Flatt suggested a pneumatic tourniquet pressure of 250 mmHg for UE applications in adults and 200 mmHg in children, a practice still widely used today [17]. More recent literature suggests using systolic blood pressure, with a safety factor of 100 mmHg added to it, but patient-related factors such as vasculopathy, obesity, and diameter of the arm are often not considered [18].

The literature evidence regarding tourniquet timing is similarly scattered. Brunner proposed a safe tourniquet time of one hour in healthy adults below middle age with reapplication of pressure after a 10-minute period of release [16]. Flatt on the other hand suggested two hours as a reasonable tourniquet time, with a 15-min deflation for the reperfusion period if this is to be exceeded [17]. A more recent study shows increased rates of tourniquet-related ischemic complications and muscle dysfunction in tourniquet times exceeding two hours [19]. On the other hand, one paper suggests that the human UE can usually tolerate prolonged compression, and that pneumatic tourniquets, with pressures as high as 300 mmHg with applications for three continuous hours, have been safely used during hand surgery [20].

In regard to the management of TRNI, the diagnosis becomes apparent only after anesthesia. Possible differential diagnoses following a loss of sensory and motor function include long-acting block, nerve injury during application of the nerve block, nerve compression due to faulty patient positioning, and/or blood pressure cuff. The approach to diagnosis and management of TRNI may be thought of as similar to a closed nerve injury without associated muscle necrosis.

**Figure 2:**
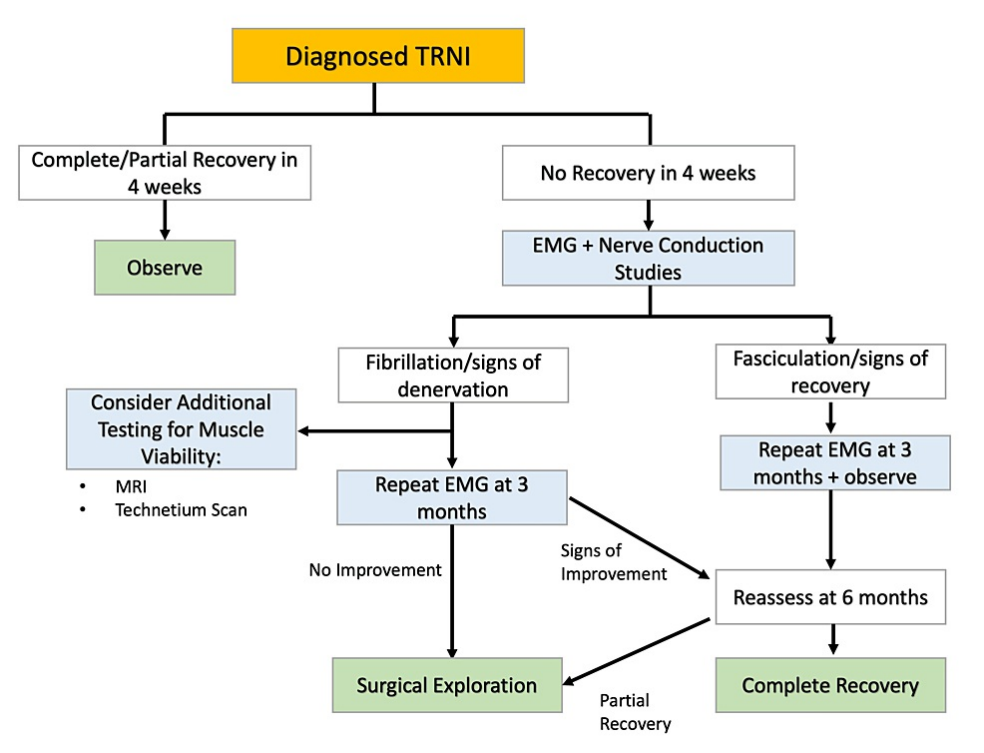
Diagram showing the algorithm of approach for management of cases with Tourniquet-Related Nerve Injury (TRNI).

There is currently no consensus on how to manage TRNI following an established diagnosis and ruling out other potential causes. In the absence of such guidelines, a novel algorithm for approaching cases with TRNI is proposed in Figure 2.

A comprehensive physical exam, including sensory and motor evaluation of appropriate areas, during the postoperative follow-up period, is prudent in establishing a diagnosis. Sensory evaluation should include two-point discrimination and reporting the sensory function based on consensus numerical scales [21-24]. Motor function is most commonly evaluated and graded based on the British Medical Research Council (MRC) grading scale [23]. Follow-up exams should occur approximately 3-4 weeks after suspected diagnosis. If at this time there is a lack of appreciable functional recovery, additional testing involving electrodiagnostic evaluation of the extremity in question should be done (i.e., EMG and NCSs) [20,22].

While electroneurography/NCSs have been described to be performed in the immediate postoperative period, these tests should be conducted at one-month intervals following the nerve injury to allow for recovery of potential physiological denervations [21,22,24]. EMG may show fibrillation which is a sign of denervation, or fasciculation which is a sign of recovery. Based on the results of electrodiagnostic testing, additional evaluation with MRI or technetium scan may be necessary to investigate muscle viability. EMG and NCSs should be repeated three to six months after the injury, at which point the presence of no improvement or only limited improvement is an indication for potential surgical intervention.

The timing of surgical intervention is a vital factor in the ability to restore function. The anatomical location of the TRNI as well as the goals of reconstruction may factor in when selecting the appropriate technique. For example, in cases of UE TRNI without recovery, the surgeon may consider the possibility of attempting neurolysis, distal nerve decompression, and even nerve transfers in cases where conservative surgical options will not be successful. The goals of UE TRNI surgical intervention include the protection of sensation and restoration of function in the hand [25]. In UE nerve transfers are favored over other methods of nerve reconstruction i.e. nerve autograft, as it can avoid harvesting a nerve graft and provide an optimal chance of functional recovery [26]. Furthermore, studies have shown similar if not better results from nerve transfer compared to that of long nerve grafts. In cases where long nerve grafts are required, our recommendation is to consider a nerve transfer and provide the patient with the highest chance of functional recovery. In the UE, there are numerous potential donors for nerve transfer including the C5 nerve root, C7 nerve root, and ulnar nerve [25].

The goals of nerve reconstruction for TRNI in LE are sensory protection and restoration of motor function most commonly to nerves controlling dorsiflexion and plantarflexion [27]. Strategies for LE nerve reconstruction can vary. In comparison to the UE nerve reconstruction, nerve transfer techniques in LE are limited, as such, in cases requiring reconstruction of nerve deficit over a large gap, nerve graft utilizing the sural nerve as a donor is frequently utilized [27]. Furthermore, based on the clinical scenario it may be necessary to consider additional tendon transfer to reestablish limb function.

The current study has its drawbacks. The study was not registered on PROSPERO nor the protocol manuscript was published prior to completion. The data available in the current literature were not uniform in terms of investigations or follow-up period. The study was done to broadly note the published management of TRNI. Further studies may be needed to evaluate specific diagnostic or treatment modalities. Finally, only surgical tourniquet application was studied and not pre-hospital or field tourniquet use. Future studies on the natural evolution of TRNI as well as comparing the TRNI in a hospital setting to TRNI in field tourniquet application may be beneficial.

## Conclusions

TRNI appears to be relatively uncommon; however, complications associated with tourniquet use, especially in the upper extremities, can be deleterious. The reported incidence of TRNI varies in the literature, and many still believe it is underreported. The most frequent scenario includes patients presenting with varying degrees of motor and sensory disturbances following a tourniquet use, which almost always resolve over the next several months.

This paper evaluates the current literature on reported cases of TRNI along with their management. We reviewed 203 total reported cases of TRNI, of which 89.7% (182/203) were found to have a complete recovery, 8.3% (17/203) had partial recovery and 2% (4/203) had no recovery reported. There is a paucity of published literature on the management of TRNI. The role and timing of EMG and NCS studies remain non-standardized but proper utilization will aid surgeons in providing patients with earlier information and reassurance about their injury and outcomes. Further investigations regarding the patient-related factors, such as limb diameter, BMI, age, and comorbidities on required tourniquet pressure and implication of TRNI are necessary. Prospective studies with a controlled setting, multivariable analysis, and large sample size are required to develop a consensus guideline for safe tourniquet application.
